# GLP-2: A POORLY UNDERSTOOD MEDIATOR ENROLLED IN VARIOUS BARIATRIC/METABOLIC SURGERY-RELATED PATHOPHYSIOLOGIC MECHANISMS

**DOI:** 10.1590/0102-6720201600040014

**Published:** 2016

**Authors:** Everton CAZZO, Martinho Antonio GESTIC, Murillo Pimentel UTRINI, Felipe David Mendonça CHAIM, Bruno GELONEZE, José Carlos PAREJA, Elinton Adami CHAIM, Daniéla Oliveira MAGRO

**Affiliations:** 1 Department of Surgery; 2 Research Laboratory in Metabology and Diabetes (Limed), State University of Campinas - Unicamp, Campinas, SP, Brazil

**Keywords:** Glucagon-like peptide 2, Bariatric surgery, Obesity, Gastric bypass, Biliopancreatic diversion.

## Abstract

**Introduction:**

Glucagon-like peptide-2 (GLP-2) is a gastrointestinal hormone whose effects are predominantly trophic on the intestinal mucosa.

**Aim:**

Critically evaluate the current literature on the influence of bariatric/metabolic surgery on the levels of GLP-2 and its potential clinical implications.

**Method:**

s: Narrative review through online research on the databases Medline and Lilacs. There were six prospective human studies, two cross-sectional human studies, and three experimental animal studies selected.

**Results:**

There is evidence demonstrating significant increase in the levels of GLP-2 following gastric bypass, Scopinaro operation, and sleeve gastrectomy. There are no differences between gastric bypass and sleeve gastrectomy in regards to the increase in the GLP-2 levels. There is no correlation between the postoperative levels of GLP-2 and the occurrence of adequate or insufficient postoperative weight loss.

**Conclusion:**

GLP-2 plays significant roles on the regulation of nutrient absorption, permeability of gut mucosa, control of bone resorption, and regulation of satiety. The overall impact of these effects potentially exerts a significant adaptive or compensatory effect within the context of varied bariatric surgical techniques.

## RESUMO

**Introdução:**

O peptídeo semelhante ao glucagon-2 (GLP-2) é hormônio gastrointestinal com efeitos predominantemente tróficos sobre a mucosa intestinal.

**Objetivo:**

Avaliar criticamente a literatura atual a respeito da cirurgia bariátrica/metabólica sobre os níveis de GLP-2 e suas potenciais implicações clínicas.

**Métodos:**

Revisão narrativa realizada através de pesquisa on-line nas bases de dados Medline e LILACS. Foram selecionados seis estudos prospectivos em humanos, dois transversais em humanos e três experimentais em animais.

**Resultados:**

Existem evidências demonstrando aumento significativo nos níveis de GLP-2 após o *bypass* gástrico, a operação de Scopinaro e a gastrectomia vertical. Não foram observadas diferenças entre o bypass gástrico e a gastrectomia vertical em relação ao aumento do GLP-2. Não há correlação entre os níveis de GLP-2 e a ocorrência de perda de peso pós-operatória adequada ou insuficiente.

**Conclusão:**

O GLP-2 desempenha importantes papel sobre a regulação da absorção de nutrientes, permeabilidade da mucosa intestinal, controle da reabsorção óssea e regulação da saciedade. O impacto combinado destes efeitos potencialmente exerce efeito adaptativo ou compensatório importante no contexto das diferentes técnicas bariátricas.

## INTRODUCTION

Glucagon-like peptide-2 (GLP-2) is a gastrointestinal hormone composed by 33 aminoacids and released from the cleavage of proglucagon in the central nervous system and the L-cells of the small and large bowels. It exerts predominantly trophic effects on the enterocytes, by means of stimulating gut cell proliferation and inhibiting crypt cell apoptosis; moreover, it enhances nutrient absorption, reduces mucosal permeability, and inhibits gut motility and gastric secretion^13^ ^,^ ^14^. The control of the intestinal expression of proglucagon and, consequently, GLP-2 is mediated by the passage of nutrients; food intake is the primary stimulus to induce the intestinal gene expression of proglucagon and the synthesis and release of proglucagon-derived gut peptides^13^ ^,^ ^14^ ^,^ ^18^. Its inactivation is performed by the enzyme dipeptil-peptidase-4 (DPP-IV) and begins within minutes after its release; after 60 minutes, only about 70% of the original GLP-2 remains intact^18^.

 The various techniques adopted in the bariatric/metabolic surgery field lead to variable changes in the production and release of gastrointestinal hormones. Some of these hormones, such as glucagon-like peptide-1 (GLP-1), ghrelin, and peptide YY (PYY) have been thoroughly studied recently, and play significant roles in the regulation of satiety and glucose metabolism after the procedures^6^ ^,^ ^25^. On the other hand, GLP-2, despite its significant effects on various physiological mechanisms related to the digestive tract and to other systems, has not been so extensively evaluated within the context of the bariatric and metabolic procedures.

This study aimed to perform a critical analysis of the existing literature on the changes caused by bariatric/metabolic surgery on the release of GLP-2 and its potential implications.

## METHODS

A narrative review of the current literature was performed through online search for the MeSH terms "glucagon-like peptide-2", "bariatric surgery", "gastric bypass", "biliopancreatic diversion" e "sleeve gastrectomy" in the databases Medline (via Pubmed) and Lilacs (via Bireme). The articles were located and analyzed, emphasizing those which reported the influence of the surgical bariatric techniques on the release of GLP-2 and discussed on its potential implications on clinical practice.

**FIGURE 1:**
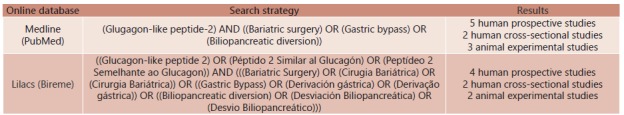
Results of the search in the online databases for the influence of bariatric/metabolic surgery on the release of GLP-2

Figure 1 presents the main results observed after the search in the online databases.

A significant overlap was observed in regard to the studies found in the both databases. Additionally, data from a study presented a Brazilian congress were also considered. At the end of the extensive research, the studies selected for data compilation were: six human prospective studies; two human cross-sectional studies; and three animal experimental studies. Since the studies evaluated different aspects on the influence of bariatric/metabolic surgery on GLP-2 release, the results will be presented in specific topics.

## RESULTS

### Gastric bypass and GLP-2 

Roux-en-Y gastric bypass is the most performed bariatric surgical procedure worldwide nowadays, accounting for more than 40% of all proceedings^1^. It is characterized by the creation of a 40-ml gastric pouch, along with a Roux-en-Y bypass with functional exclusion of gastric remnant, duodenum and proximal jejunum, with a 100 cm excluded biliopancreatic limb and a Roux limb with about 150 cm; common channel comprehends the remaining intestine^7^ ^,^ ^15^ It is associated with significant resolution rates of obesity-related comorbidities and long-term maintenance of excess weight loss^5^.

As it occurs with GLP-1, it has also been observed a significant increase in the expression and secretion of GLP-2 following this technique. Taqi et al.^29^, in an experimental study, demonstrated a significant increase in the GLP-2 levels after gastric bypass in rats. LeRoux et al.^23^, in a human prospective study, demonstrated a significant increase in the postprandial levels of GLP-2 after gastric bypass, with a secretion peak observed six months after the procedure. Jacobsen et al.^22^, in a human prospective study, observed significant increase in the postprandial levels of GLP-2 two weeks after gastric bypass. Cazzo et al.^8^, in a human prospective study, observed a significant increase in the GLP-2 levels 12 months after surgery, and demonstrated that this increase was significantly correlated with aspects of satiety regulation. A cross-sectional study conducted by DeHollanda et al.^11^, which evaluated individuals who underwent gastric bypass at the 24^th^ postoperative month, did not observe statistically significant differences between the levels of GLP-2 of individuals with adequate weight loss compared to other with failed weight loss. Another cross-sectional study, conducted by Valderas et al.^30^, observed significantly higher postprandial levels of GLP-2 in postmenopausal women who underwent gastric bypass when compared with non-operated controls. The main hypotheses to explain the postoperative increase of GLP-2 are the functional exclusion of the duodenum (foregut hypothesis) and the passage of a greater volume of nutrients by the distal small bowel (hindgut hypothesis). Hence, there would be a greater stimulus for the expression and release of GLP-2 in the L-cells of the distal small bowel^32^.

### Biliopancreatic diversions and GLP-2 

There are two surgical procedures classified as biliopancreatic diversion: the Scopinaro operation and the duodenal switch. The Scopinaro operation, considered the classic biliopancreatic diversion, is characterized by a distal gastrectomy with maintenance of a 300-ml gastric pouch, along with a long gastroenteric bypass, with a 50-80 cm common limb^28^. The duodenal switch operation was conceived as a modification of the Scopinaro operation which aimed to improve gastric emptying and reduce the postoperative occurrence of diarrhea and malabsorption; it is characterized by a sleeve gastrectomy with resection of the greater curvature of the stomach, along with a Roux-en-Y duodenal-enteric bypass with a 80-150 cm common limb^3^. Both procedures are associated with the highest rates of resolution of diabetes mellitus among all other bariatric techniques; however, they are also associated with the occurrence of protein-calorie malnutrition^5^. In the last registry report of the International Federation for the Surgery of Obesity and Metabolic Disorders (IFSO), the duodenal switch accounted for 1.5% of all bariatric proceedings performed worldwide, whereas the Scopinaro operation accounted for less than 1%^1^.

These procedures lead to functional exclusion of duodenum and proximal jejunum, and to the passage of greater volumes of nutrients through even more distal portions of the small bowel; consequently, there is an even higher stimulus for the GLP-2 expression in the L-cells of the terminal ileum and colon. A pioneering study by Borg et al.^4^ demonstrated a significant increase in the levels of GLP-2 after the Scopinaro operation in rats. This finding was confirmed in humans by Cazzo et al.^9^ in a prospective study.

### Sleeve gastrectomy and GLP-2

The sleeve gastrectomy, at first developed as the first part of the two-stage duodenal switch performed in high-risk individuals, proved to be efficient to lead to weight loss and resolution of comorbidities when solely performed. It is characterized by the resection of the gastric greater curvature and creation of a tubular stomach through calibration with a bougie^19^. To date, it is the second most performed bariatric surgical procedure worldwide, accounting almost 40% of all proceedings^1^.

Comparing individuals who underwent gastric bypass and sleeve gastrectomy, Romero et al.^27^ observed, in a prospective study, that both procedures led to significant increase in the postprandial levels of GLP-2 six weeks after surgery, without significant difference between the two evaluated procedures. Cummings et al.^10^, in an experimental study, demonstrated a significant increase in the GLP-2 levels in rats after the sleeve gastrectomy. Once this procedure is exclusively based in gastric resection, there is no consensus on its incretin-secretion potential. Ghrelin, a peptide released in the gastric fundus and related to satiety regulation, has its secretion affected by the surgery; however, in regard to glucagon-like peptides, significant changes were not expected, since there is no duodenal exclusion or bypass to the distal intestine. Nonetheless, there is consistent evidence of the increase in the GLP-2 levels after this procedure. There is the theory that this effect might be related to changes in the gastric emptying speed caused by the tubular form of the stomach^24^.

The studies included in this section, as well as their designs and main results, are summarized in Table 1.

**TABLE 1:** Main studies included in this review

Study	Methods	Procedure	Follow-up	Results
Taqi et al. [10]	Animal experimental	*Gastric bypass*	14 days	Significant increase of GLP-2
LeRoux et al. [11]	Human prospective	*Gastric bypass*	6 months	Significant increase of GLP-2
Jacobsen et al. [12]	Human prospective	*Gastric bypass*	14 days	Significant increase of GLP-2
Cazzo et al. [13]	Human prospective	*Gastric bypass*	12 months	Significant increase of GLP-2
DeHollanda et al. [14]	Human cross-sectional	*Gastric bypass*	N.A.	Similar levels of GLP-2 in individuals with adequate and insufficient weight loss
Valderas et al. [15]	Human cross-sectional	*Gastric bypass*	N.A.	Higher levels of GLP-2 in operated individuals
Borg et al. [19]	Animal experimental	Scopinaro	23 days	Significant increase of GLP-2
Cazzo et al. [20]	Human prospective	Scopinaro	12 months	Significant increase of GLP-2
Romero et al. [22]	Human prospective	*Gastric bypass and Sleeve gastrectomy*	6 months	Significant increase of GLP-2 in both procedures
Cummings et al. [23]	Animal experimental	Sleeve gastrectomy	4 months	Significant increase of GLP-2

## DISCUSSION

GLP-2 presents physiological effects that, at first glance, did not cause so much interest in the bariatric/metabolic surgery field as GLP-1, which is strongly related to the early improvement in the insulin sensitivity observed after varied procedures. However, when analyzed in a wider fashion, the several properties of GLP-2 may contribute to the metabolic equilibrium of the individuals who undergo bariatric surgery in such an adaptive and compensatory way, since it is able to minimize potential harms that may be caused by these procedures.

The trophic effect on the gut mucosa played by GLP-2 is considered to be its prime property^13^ ^,^ ^18^. The exogenous application and the increased release after surgery are associated with gut mucosal hypertrophy and increase in the nutrient absorption capacity, as it has been previously demonstrated in rats^4^. It is arguable that the increase in the absorption capacity might be a compensatory mechanism within the bariatric context, significant for the stabilization of the achieved weight loss and reducing the potential risk of late malnutrition among the operated individual, especially those who underwent biliopancreatic diversions. Furthermore, this GLP-2 effect possibly might be related to the decrease in the intensity of diarrhea and fat malabsorption observed in the late postoperative phase of the biliopancreatic diversions^16^.

In procedures which comprise exclusion of long intestinal segments, there is the possibility of bacterial overgrowth, a factor that is proved to be associated with higher release of lipopolysaccharides and endotoxemia. Another proven effect of GLP-2 is the reduction of the gut mucosa permeability in rats, which occurs by means of transcellular and paracellular mechanisms. Hence, the increase observed in its secretion may present significant protective effect in individuals who underwent distal intestinal bypasses, preventing the occurrence of endotoxemia e decreasing the risk of harmful effects, especially on the liver^2^ ^,^ ^17^.

The occurrence of bone mineral metabolism disturbances is common after bariatric surgery; there is a significant increase in the bone demineralization, secondary hyperparathyroidism, urinary stones, and osteopenia/osteoporosis among operated individuals. There is evidence that the exogenous application of GLP-2 reduces the bone resorption in the postprandial period, and also decreases nightly bone resorption, even leading to a slight increase in the bone mineral density^20^ ^,^ ^21^.

Amongst the mechanisms enrolled in weight loss after bariatric procedures, changes in the satiety regulation may exert significant effects. There is evidence of a significant correlation between the higher levels of GLP-2 after gastric bypass and a subjective change in specific aspects of satiety, related to satiation feeling and the desire to eat, in a different manner than that exerted by GLP-1, which might be more related to the hunger feeling^13^.

The scarce volume of evidence, especially in humans, evaluating the effects of bariatric surgery on GLP-2 is a limiting factor for ultimate conclusions in regard to the correlation between the procedures and the hormone release, as well as the interplay of both. Nonetheless, it must be emphasized that the body of evidence available to date permits to conclude that there is a strong possibility that the increase in the levels of GLP-2 observed after varied bariatric surgical techniques may play several roles on the homeostasis process, not only in an adaptive and compensatory fashion, but also related to sacietogen-incretin mechanisms enrolled in the postoperative metabolic balance. The necessity for further research on this theme must also be emphasized, leading to a wider and ultimate understanding.

## CONCLUSION

The currently available evidence on the influence of bariatric/metabolic surgery on GLP-2 demonstrates that there is a postoperative increase in the levels of this hormone, and this change may be potentially related to weight loss stabilization, late reduction of diarrhea and malabsorption, partial compensation of harms to bone mineral metabolism, minimization of the consequences of bacterial overgrowth, and regulation of specific aspects of satiety regulation.
